# Publisher Correction: Metabolic characteristics of CD8^+^ T cell subsets in young and aged individuals are not predictive of functionality

**DOI:** 10.1038/s41467-020-17441-9

**Published:** 2020-07-09

**Authors:** Kylie M. Quinn, Tabinda Hussain, Felix Kraus, Luke E. Formosa, Wai K. Lam, Michael J. Dagley, Eleanor C. Saunders, Lisa M. Assmus, Erica Wynne-Jones, Liyen Loh, Carolien E. van de Sandt, Lucy Cooper, Kim L. Good-Jacobson, Katherine Kedzierska, Laura K. Mackay, Malcolm J. McConville, Georg Ramm, Michael T. Ryan, Nicole L. La Gruta

**Affiliations:** 1 Department of Biochemistry and Molecular Biology, Monash Biomedicine Discovery Institute, Monash University, Clayton, VIC 3800 Australia; 2 School of Health and Biomedical Sciences, RMIT University, Bundoora, VIC Australia; 3 Department of Biochemistry and Molecular Biology, Bio21 Institute of Molecular Science and Biotechnology, University of Melbourne, Parkville, VIC 3010 Australia; 4 Institute of Experimental Immunology, University Hospital Bonn, 53127 Bonn, Germany; 5 Department of Microbiology and Immunology, University of Melbourne, Peter Doherty Institute for Infection and Immunity, Parkville, VIC Australia; 6 Department of Hematopoiesis, Sanquin Research and Landsteiner Laboratory, Amsterdam UMC, University of Amsterdam, 1066CX Amsterdam, Netherlands; 7 Present Address: Monash Ramaciotti Centre for Cryo-EM, Monash University, Clayton, VIC Australia

**Keywords:** Lymphocyte activation, Cytotoxic T cells

Correction to: *Nature Communications* 10.1038/s41467-020-16633-7, published online 5 June 2020.

In the original version of this Article, Author Kylie M. Quinn, was incorrectly assigned a present address.

This error has been corrected in both the PDF and HTML versions of the Article.

