# Supplementary material for: Metabolic characteristics of CD8+ T cell subsets in young and aged individuals are not predictive of functionality
Source: Nat Commun. 2020 Jun 5;11:2857. doi: 10.1038/s41467-020-16633-7 (PMC7275080; doi:10.1038/s41467-020-16633-7)
Supplement: Supplementary file 1 — Supplementary Information [file 41467_2020_16633_MOESM1_ESM.pdf]

Supplementary Information Cover Page

Metabolic Characteristics of Young and Aged CD8$^+$ T cell Subsets Do Not Predict Functionality

Quinn et al

Manuscript No. NCOMMS-19-23928-T

Supplementary Figure 1:

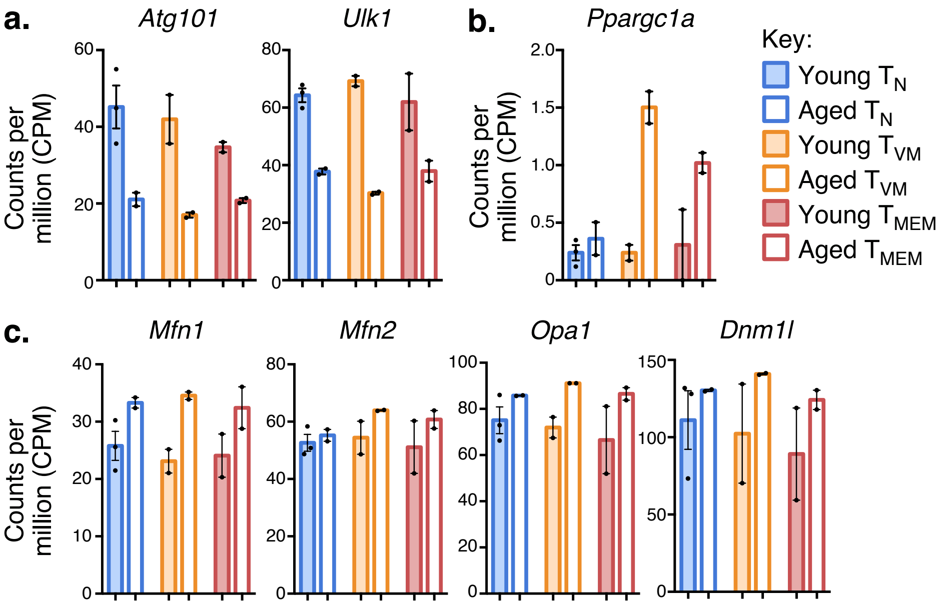

Supplementary Figure 1. Analysis of transcripts involved in mitophagy, mitochondrial biogenesis, fusion, and fission. Shown are the relative transcript levels of **a**, *Atg101* and *Ulk1*, **b,** *Ppargc1a*, and **c**, *Mfn1*, *Mfn2*, *Opa1*, and *Dnm1l* from RNA-Seq data[17] from T_N, T_{MEM}, and T_{VM} cells sorted and analysed *ex vivo* from naïve young and aged SPF mice. Shown is mean ± standard error of the mean (SEM).

Supplementary Figure 2:

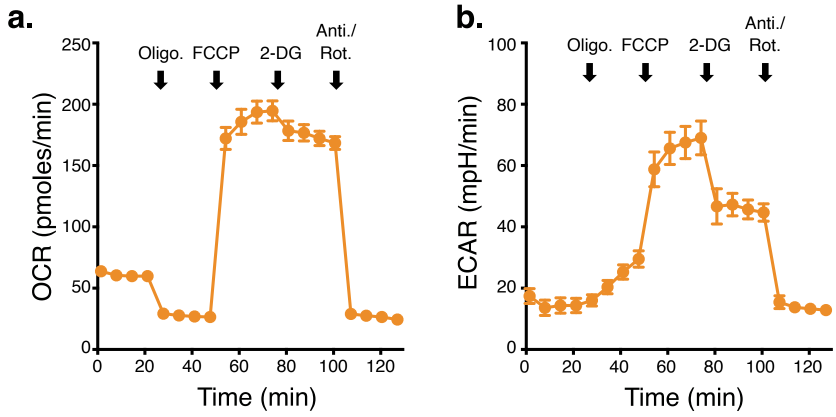

Supplementary Figure 2. Impact of glycolysis on SRC in TVM cells. **a,** OCR for sorted TVM cells from the spleens of uninfected young SPF mice and **b,** ECAR for the same cells, with the addition of 2-DG indicated (n=2, 2 experimental replicates).
